# Navigating Policy Shifts in Home Health: Implications for Care, Quality, and High-Need Populations

**DOI:** 10.1093/geroni/igaf122.281

**Published:** 2025-12-31

**Authors:** Jamie Smith, Jacy Weems, Shekinah Fashaw-Walters

## Abstract

This symposium focuses on the evolving landscape of home health care (HH) and the impact of key federal and state-level policies on HH delivery. The HH setting has historically been highly responsive to regulatory shifts and policy nudges. As we analyze the effects of the new payment model, the Patient-Driven Groupings Model (PDGM), state-level Certificate of Need laws, and prioritization of quality ratings, we must understand that HH patients are not a homogeneous group. HH patients have different care needs, often driven by their health, functional status, and at-home support. This symposium features four studies that explore the impact of various policies on HH care. Two presentations focus specifically on PDGM and examine how certain aspects of the model influence HH service utilization. The first presentation examines how PDGM has impacted community-entry HH use among individuals living with dementia. The second presentation investigates changes in post-acute therapy services for older adults transitioning home following hospitalization. The third presentation examines the provision of high-quality HH for individuals with dementia, raising questions about the effectiveness of current measures in capturing care quality. The fourth presentation evaluates the impact of Certificate of Need laws on HH use among persons with Alzheimer’s disease and related dementias. These innovative studies will foster nuanced discussions about the complexities of the current HH environment and inform future policy recommendations.

